# Freezing of gait and response conflict in Parkinson's disease: computational directions

**DOI:** 10.3389/fncom.2014.00007

**Published:** 2014-01-28

**Authors:** Ahmed A. Moustafa

**Affiliations:** Marcs Institute for Brain and Behaviour and School of Social Sciences and Psychology, University of Western SydneySydney, NSW, Australia

**Keywords:** Parkinson's disease, freezing of gait, response conflict, computational modeling

In a recent study, Matar et al. (2013) found that Parkinson's disease (PD) patients with freezing of gait significantly displayed reduced locomotive responses when passing through narrow rather than wide doors and while facing the opening of a sliding door. Freezing of gait refers to a cessation of movement despite the intention to walk forward, such that patients often feel like their feet have been “*glued to the ground*” (Schaafsma et al., 2003; Rahman et al., 2008). This pattern of results was not found with PD patients without freezing of gait or healthy controls. The Matar et al. study can potentially point to which environmental situations can lead to freezing of gait in PD patients.

**Figure 1:**
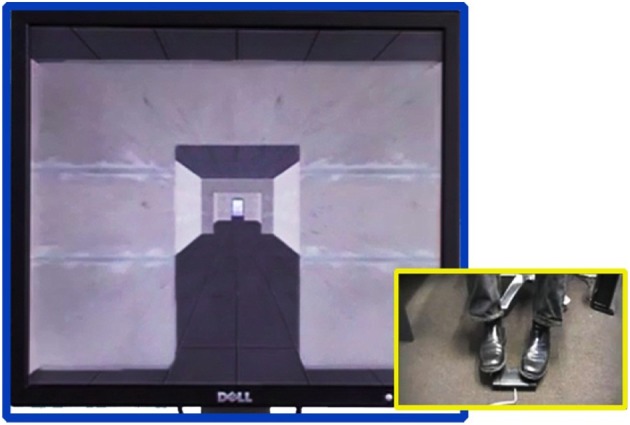
**Virtual reality locomotion paradigm used in the Matar et al. (2013) study**. The right side shows feet resting over a button box to control and measure the stepping action of a subject while reacting to stimuli appearing in the virtual reality environment (left). The paradigm allows for measuring accuracy (i.e., whether the subject performs correct movements) and latency of foot movement (i.e., time taken to initiate walking response) while the subject is navigating the environment via locomotive responses and alternating left–right sequences of footsteps.

Most interestingly, these results were reported by using a “virtual” setting in which subjects navigate a realistic three-dimensional environment using foot pedals (see Figure 1, also see Naismith and Lewis, 2010). The virtual reality paradigm used in the Matar et al. (2013) study is used for testing perceptual and cognitive factors underlying successful locomotion in humans. In this paradigm, forward progression in the virtual environment (left side of Figure 1) only occurs by alternating left–right sequences of footsteps (right side of Figure 1). The time taken between the footsteps (left-right or right-left) corresponds to faster or slower progression in the virtual environment, thus mimicking natural walking. In the Matar et al. study, the virtual environment contains corridors with doorways (as shown in Figure 1) and/or salient environmental stimuli that prompt locomotive responses. The Matar et al. data confirms and also extends prior results that have used real doorways (Almeida and Lebold, 2010; Cowie et al., 2010; Knobl et al., 2012).

The virtual reality paradigm also allows the testing of more abstract cognitive cues on freezing of gait. Interestingly, the same study by Matar et al. also show that subjects with freezing of gait (but not other groups) slow down when instructed to “walk” when presented with word “RED” in red-font rather than “GREEN” in green font (Matar et al., 2013). Although non-significant, healthy controls took longer time to respond to the “RED” than “GREEN” cue. The authors suggested that the red cue is implicitly associated with stopping action (e.g., red-light traffic signals), and thus instructing subjects to walk when presenting with the red cue can lead to response conflict (e.g., conflict resulting from deciding whether to walk quickly, slowly, or stop). The authors further argue that PD patients with freezing of gait have a response conflict processing deficit, which leads to slow locomotion during the presentation of the red cue.

It is argued that an impaired response conflict mechanism can explain the freezing of gait phenomenon (Matar et al., 2013; Shine et al., 2013). For example, Matar et al. argue that the same response conflict mechanism can also explain the differential effects of doorway size on locomotive speed: While passing through the narrow door, subjects might hit the wall, and will think about various other motor responses, which might induce response conflict. The same mechanism also explains reduced locomotive speed while facing a sliding door. Interestingly, the same response conflict mechanism can also explain prior studies showing that avoiding obstacles (Snijders et al., 2010) or making a turn (Spildooren et al., 2010) might lead to freezing of gait in PD patients.

What is the neural mechanism underlying freezing of gait and response conflict? Shine et al. (2013) found that PD patients with freezing of gait show aberrant neural activation in the pre-supplementary motor area and subthalamic nucleus (STN) in situations that involve choosing either to walk or stop. Along the same lines, Frank et al. (2007) found that PD patients tested on STN DBS respond faster in conflicting situations than the same patients tested off their STN DBS, suggesting that STN plays a role in the time taken to generate a motor response. Most prior studies of the role of the STN in response conflict employed hand movements (Frank et al., 2007; Isoda and Hikosaka, 2008); however, it is not known if the STN plays a similar role in locomotion (i.e., whether the STN controls timing of locomotive responses). For a recent review for the role of STN in high-conflict decision making, see Weintraub and Zaghloul (2013).

Future computational network modeling research is needed to tie together behavioral and neural data regarding the occurrence of freezing of gait in Parkinson's disease. The importance of these models is to explain seemingly different phenomena, such as the relationship between freezing of gait and response conflict, as well as the role of STN in these processes. Such models can provide a mechanistic account for the role of cortex-basal ganglia interactions, and can also simulate the dissociable effects the dopamine medications and deep brain stimulation on freezing of gait. For example, computational models by Frank and colleagues can potentially explain the occurrence of freezing of gait in Parkinson's disease patients (Frank, 2006; Frank et al., 2007). In these models, the STN acts as a global inhibition mechanism, such as to inhibit motor processes during high-conflict situations. The model suggests freezing of gait occurs in high-conflict situation due the over activation of the STN, which leads to inhibition of motor output and thus freezing of gait.

Future experimental research should also test the dissociable effects of different dopaminergic therapies (e.g., levodopa, dopamine agonists, MAO inhibitors) on freezing of gait in relation to environmental contexts in which it commonly occurs. The success of the virtual reality paradigm to reveal perceptual and cognitive factors underlying freezing behavior will open venues for studying more complex paradigms in which locomotive responses may be disrupted, such as while driving or crossing the street.
